# Supplementary material for: A Critical View of the Application of the APEX Software (Aqueous Photochemistry of Environmentally-Occurring Xenobiotics) to Predict Photoreaction Kinetics in Surface Freshwaters
Source: Molecules. 2019 Dec 18;25(1):9. doi: 10.3390/molecules25010009 (PMC7017383; doi:10.3390/molecules25010009)
Supplement: Supplementary file 1 [file molecules-25-00009-s001.zip › 3.2.4_gcc-4.4.0.zip!/3.2.4_gcc-4.4.0/3.2.4_gcc-4.4.0/doc/gnuplot/postscript-terminal/ps_fontfile_doc.pdf]

Using TeX Fonts in the Gnuplot Postscript Terminal

Harald Harders, h.harders@tu-bs.de

2003-03-03

The Postscript terminal can embed Postscript Type 1 fonts (with extensions `.pfa` and `.pfb`) and TrueType fonts (extension `.ttf`)[1] using the command

```
set terminal postscript fontfile '<filename>'
```

The `fontfile` option can be used multiple times. See the sections *set terminal postscript* and *set fontpath* in the Gnuplot documentation for further description.

The embedded font can be used by

```
set terminal postscript '<fontname>' <size>
```

or in postscript enhanced terminal as following example:

```
set xlabel '{/CMMI10 x}'
```

Among other things, the font embedding is useful for generating plots to be included in LaTeX documents. For normal text, the *cm-super* Postscript Type 1 fonts are a good choice. They are available from CTAN servers, e.g.

```
ftp://ftp.dante.de/tex-archive/fonts/ps-type1/cm-super/
```

The normal upright font with serifes is defined in `sfrm1000.pfb`, and the font name is `SFRM1000`[2] (The `1000` means that this font is designed for 10 pt). Replace the `rm` by `it`, `bx` or other combinations in both the file name and the font name (here, in uppercase letters) in order to get other font shapes. The encoding of these fonts is ordinary and thus is not described here. Table 1 shows some examples of fonts contained in the cm-super font bundle.

For mathematics the Type 1 versions of the Computer Modern fonts are useful. They should be installed in most TeX implementations and are also available from CTAN servers, e.g.

```
ftp://ftp.dante.de/tex-archive/fonts/cm/ps-type1/bluesky/pfb/
```

Here, the font name is the base of the file name in uppercase letters, e.g. the file `cmmi10.pfb` contains the font `CMMI10`. Since the encoding of these fonts is strange, a table containing all characters for some fonts follows. The font `CMEX10` contains large symbols for mathematics. They overlap sometimes in the table. Since the baseline of the `CMEX10` font is at the top of the signs, Gnuplot defines a font `CMEX10-Baseline` with a different baseline if `CMEX10` is embedded (normally by using `fontfile 'cmex10.pfb'`. In contrast to the other fonts, `CMEX10` is only available in the design size 10 pt.

You can access all characters of the fonts by typing their octal code. To get a ♡ symbol, you may type:

```
set label '{/CMSY10 \176}' at graph 0.5,0.5
```

[1] If `.pfb` and `.ttf` fonts really can be embedded depends on your gnuplot installation: It needs to be able to handle pipes.

[2] If you have an old version of the cm-super font, prior 2001-10-14, the font name is in lowercase letters: `sfrm1000`. You should update to a new version.

Table 1: Some fonts in the cm-super font bundle (for a designsize of 10 pt)

File name	Full font name (all preceded by `Computer Modern`)	Example
`sfrm1000.pfb`	`Roman`	Example
`sfbx1000.pfb`	`Bold Extended`	**Example**
`sfti1000.pfb`	`Italic`	*Example*
`sfbi1000.pfb`	`Bold Extended Italic`	***Example***
`sfsl1000.pfb`	`Slanted`	*Example*
`sfbl1000.pfb`	`Bold Extended Slanted`	***Example***
`sfcc1000.pfb`	`Caps and Small Caps`	EXAMPLE
`sfss1000.pfb`	`Sans Serif`	Example
`sfsi1000.pfb`	`Sans Serif Slanted`	*Example*
`sfsx1000.pfb`	`Sans Serif Bold Extended`	**Example**
`sfso1000.pfb`	`Sans Serif Bold Extended Slanted`	***Example***
`sftt1000.pfb`	`Typewriter`	`Example`
`sfit1000.pfb`	`Typewriter Italic`	*`Example`*
`sfst1000.pfb`	`Typewriter Slanted`	*`Example`*
`sftc1000.pfb`	`Typewriter Caps and Small Caps`	`EXAMPLE`

Since characters with an octal number below \040 can't be displayed by some postscript interpreters, these characters are repeated in the Computer Modern Fonts with a larger code. Thus, you should use the larger number, where two octal numbers are given (e.g. \000, \241). For example, you better use

```
set xlabel '{/CMR10 \242}'
```

than

```
set xlabel '{/CMR10 \001}'
```

to get an upright uppercase Delta Δ.

Oct	CMR10	CMTI10	CMTT10	CMMI10	CMU10	CMSS10	CMTEX10	CMFF10	CMSY10	LASY10	CMEX10-Baseline	Oct	Dec
\000, \241	Γ	*Γ*	Γ	*Γ*	Γ	Γ	·	Γ	−		(	\000, \241	0, 161
\001, \242	Δ	*Δ*	Δ	*Δ*	Δ	Δ	↓	Δ	·	◁	)	\001, \242	1, 162
\002, \243	Θ	*Θ*	Θ	*Θ*	Θ	Θ	α	Θ	×	⊴	[	\002, \243	2, 163
\003, \244	Λ	*Λ*	Λ	*Λ*	Λ	Λ	β	Λ	∗	▷	]	\003, \244	3, 164
\004, \245	Ξ	*Ξ*	Ξ	*Ξ*	Ξ	Ξ	∧	Ξ	÷	⊵	⌊	\004, \245	4, 165
\005, \246	Π	*Π*	Π	*Π*	Π	Π	¬	Π	⋄		⌋	\005, \246	5, 166
\006, \247	Σ	*Σ*	Σ	*Σ*	Σ	Σ	∈	Σ	±		⌈	\006, \247	6, 167
\007, \250	Υ	*Υ*	Υ	*Υ*	Υ	Υ	π	Υ	∓		⌉	\007, \250	7, 168
\010, \251	Φ	*Φ*	Φ	*Φ*	Φ	Φ	λ	Φ	⊕		{	\010, \251	8, 169

Oct	CMR10	CMTI10	CMTT10	CMMI10	CMU10	CMSS10	CMTEX10	CMFF10	CMSY10	LASY10	CMEX10-Baseline		Oct	Dec
\011, \252	Ψ	Ψ	Ψ	Ψ	Ψ	Ψ	γ	Ψ	⊖		}		\011, \252	9, 170
\012, \255	Ω	Ω	Ω	Ω	Ω	Ω	δ	Ω	⊗			⟨	\012, \255	10, 173
\013, \256	ff	ff	↑	α	ff	ff	↑	ff	⊘		⟩		\013, \256	11, 174
\014, \257	fi	fi	↓	β	fi	fi	±	fi	⊙			∣	\014, \257	12, 175
\015, \260	fl	fl	'	γ	fl	fl	⊕	fl	◯		∥		\015, \260	13, 176
\016, \261	ffi	ffi	¡	δ	ffi	ffi	∞	ffi	∘			/	\016, \261	14, 177
\017, \262	ffl	ffl	¿	ϵ	ffl	ffl	∂	ffl	•		\		\017, \262	15, 178
\020, \263	ı	ı	ı	ζ	ı	ı	⊂	ı	≍			(	\020, \263	16, 179
\021, \264	ȷ	ȷ	ȷ	η	ȷ	ȷ	⊃	ȷ	≡		)		\021, \264	17, 180
\022, \265	`	`	`	θ	`	`	∩	`	⊆			(	\022, \265	18, 181
\023, \266	´	´	´	ι	´	´	∪	´	⊇		)		\023, \266	19, 182
\024, \267	ˇ	ˇ	ˇ	κ	ˇ	ˇ	∀	ˇ	≤			[	\024, \267	20, 183
\025, \270	˘	˘	˘	λ	˘	˘	∃	˘	≥		]		\025, \270	21, 184
\026, \271	¯	¯	¯	μ	¯	¯	⊗	¯	⪯			[	\026, \271	22, 185
\027, \272	˚	˚	˚	ν	˚	˚	⇆	˚	⪰		]		\027, \272	23, 186
\030, \273	¸	¸	¸	ξ	¸	¸	←	¸	∼			[	\030, \273	24, 187
\031, \274	ß	ß	ß	π	ß	ß	→	ß	≈		]		\031, \274	25, 188
\032, \275	æ	æ	æ	ρ	æ	æ	≠	æ	⊂			{	\032, \275	26, 189
\033, \276	œ	œ	œ	σ	œ	œ	◇	œ	⊃		}		\033, \276	27, 190
\034, \277	ø	ø	ø	τ	ø	ø	≤	ø	≪			⟨	\034, \277	28, 191
\035, \300	Æ	Æ	Æ	υ	Æ	Æ	≥	Æ	≫		⟩		\035, \300	29, 192
\036, \301	Œ	Œ	Œ	ϕ	Œ	Œ	≡	Œ	≺			/	\036, \301	30, 193
\037, \302	Ø	Ø	Ø	χ	Ø	Ø	∨	Ø	≻		\		\037, \302	31, 194
\040, \303	´	´	␣	ψ	´	´		´	←			(	\040, \303	32, 195
\041	!	!	!	ω	!	!	!	!	→		)		\041	33
\042	”	”	"	ε	”	”	"	"	↑				\042	34
\043	#	#	#	ϑ	#	#	#	#	↓				\043	35
\044	$	£	$	ϖ	£	$	$	$	↔				\044	36
\045	%	%	%	ϱ	%	%	%	%	↗				\045	37
\046	&	&	&	ς	&	&	&	&	↘				\046	38
\047	’	’	'	φ	’	'	'	'	≃				\047	39
\050	(	(	(	↼	(	(	(	(	⇐	‹			\050	40
\051	)	)	)	↽	)	)	)	)	⇒	›			\051	41
\052	*	*	*	⇀	*	*	*	*	⇑	ˆ			\052	42
\053	+	+	+	⇁	+	+	+	+	⇓	ˇ			\053	43
\054	,	,	,	‘	,	,	,	,	⇔				\054	44
\055	-	-	-	’	-	-	-	-	↖				\055	45
\056	.	.	.	▷	.	.	.	.	↙				\056	46
\057	/	/	/	◁	/	/	/	/	∝				\057	47

Oct	CMR10	CMTI10	CMTT10	CMMI10	CMU10	CMSS10	CMTEX10	CMFF10	CMSY10	LASY10	CMEX10-Baseline		Oct	Dec
\060	0	*0*	0	0	0	0	0	0	′	℧		⎛	\060	48
\061	1	*1*	1	1	1	1	1	1	∞	⋈	⎞		\061	49
\062	2	*2*	2	2	2	2	2	2	∈	□		⎡	\062	50
\063	3	*3*	3	3	3	3	3	3	∋	◇	⎤		\063	51
\064	4	*4*	4	4	4	4	4	4	△			⎣	\064	52
\065	5	*5*	5	5	5	5	5	5	▽		⎦		\065	53
\066	6	*6*	6	6	6	6	6	6	/			⎢	\066	54
\067	7	*7*	7	7	7	7	7	7	ˈ		⎥		\067	55
\070	8	*8*	8	8	8	8	8	8	∀			⎧	\070	56
\071	9	*9*	9	9	9	9	9	9	∃		⎫		\071	57
\072	:	*:*	:	.	:	:	:	:	¬	∼		⎩	\072	58
\073	;	*;*	;	,	;	;	;	;	∅	⇝	⎭		\073	59
\074	¡	*¡*	<	<	¡	¡	<	¡	ℜ	⊏		⎨	\074	60
\075	=	=	=	/	=	=	=	=	ℑ	⊐	⎬		\075	61
\076	¿	*¿*	>	>	¿	¿	>	¿	⊤			⎪	\076	62
\077	?	*?*	?	⋆	?	?	?	?	⊥		⏐		\077	63
\100	@	*@*	@	∂	@	@	@	@	ℵ			⎝	\100	64
\101	A	*A*	A	*A*	A	A	A	A	$\mathcal{A}$		⎠		\101	65
\102	B	*B*	B	*B*	B	B	B	B	$\mathcal{B}$			⎜	\102	66
\103	C	*C*	C	*C*	C	C	C	C	$\mathcal{C}$		⎟		\103	67
\104	D	*D*	D	*D*	D	D	D	D	$\mathcal{D}$			⟨	\104	68
\105	E	*E*	E	*E*	E	E	E	E	$\mathcal{E}$		⟩		\105	69
\106	F	*F*	F	*F*	F	F	F	F	$\mathcal{F}$			⨆	\106	70
\107	G	*G*	G	*G*	G	G	G	G	$\mathcal{G}$		⨆		\107	71
\110	H	*H*	H	*H*	H	H	H	H	$\mathcal{H}$			∮	\110	72
\111	I	*I*	I	*I*	I	I	I	I	$\mathcal{I}$		∮		\111	73
\112	J	*J*	J	*J*	J	J	J	J	$\mathcal{J}$			⨀	\112	74
\113	K	*K*	K	*K*	K	K	K	K	$\mathcal{K}$		⨀		\113	75
\114	L	*L*	L	*L*	L	L	L	L	$\mathcal{L}$			⨁	\114	76
\115	M	*M*	M	*M*	M	M	M	M	$\mathcal{M}$		⨁		\115	77
\116	N	*N*	N	*N*	N	N	N	N	$\mathcal{N}$			⨂	\116	78
\117	O	*O*	O	*O*	O	O	O	O	$\mathcal{O}$		⨂		\117	79
\120	P	*P*	P	*P*	P	P	P	P	$\mathcal{P}$			∑	\120	80
\121	Q	*Q*	Q	*Q*	Q	Q	Q	Q	$\mathcal{Q}$		∏		\121	81
\122	R	*R*	R	*R*	R	R	R	R	$\mathcal{R}$			∫	\122	82
\123	S	*S*	S	*S*	S	S	S	S	$\mathcal{S}$		⋃		\123	83
\124	T	*T*	T	*T*	T	T	T	T	$\mathcal{T}$			⋂	\124	84
\125	U	*U*	U	*U*	U	U	U	U	$\mathcal{U}$		⨄		\125	85
\126	V	*V*	V	*V*	V	V	V	V	$\mathcal{V}$			⋀	\126	86

Oct	CMR10	CMTI10	CMTT10	CMMI10	CMU10	CMSS10	CMTEX10	CMFF10	CMSY10	LASY10	CMEX10-Baseline	Oct	Dec
\127	W	*W*	W	*W*	W	W	W	W	𝒲		⋁	\127	87
\130	X	*X*	X	*X*	X	X	X	X	𝒳		∑	\130	88
\131	Y	*Y*	Y	*Y*	Y	Y	Y	Y	𝒴		∏	\131	89
\132	Z	*Z*	Z	*Z*	Z	Z	Z	Z	𝒵		∫	\132	90
\133	[	*[*	[	♭	[	[	[	[	∪		⋃	\133	91
\134	“	*“*	\	♮	“	“	\	“	∩		⋂	\134	92
\135	]	*]*	]	♯	]	]	]	]	⊎		⨄	\135	93
\136	ˆ	ˆ	^	⌣	ˆ	ˆ	^	ˆ	∧		⋀	\136	94
\137	˙	˙	_	⌢	˙	˙	_	˙	∨		⋁	\137	95
\140	‘	‘	‘	ℓ	‘	‘	‘	‘	⊢		∐	\140	96
\141	a	*a*	a	*a*	a	a	a	a	⊣		∐	\141	97
\142	b	*b*	b	*b*	b	b	b	b	⌊		ˆ	\142	98
\143	c	*c*	c	*c*	c	c	c	c	⌋		ˆ	\143	99
\144	d	*d*	d	*d*	d	d	d	d	⌈		ˆ	\144	100
\145	e	*e*	e	*e*	e	e	e	e	⌉		˜	\145	101
\146	f	*f*	f	*f*	f	f	f	f	{		˜	\146	102
\147	g	*g*	g	*g*	g	g	g	g	}		˜	\147	103
\150	h	*h*	h	*h*	h	h	h	h	⟨		[	\150	104
\151	i	*i*	i	*i*	i	i	i	i	⟩		]	\151	105
\152	j	*j*	j	*j*	j	j	j	j	|		⌊	\152	106
\153	k	*k*	k	*k*	k	k	k	k	‖		⌋	\153	107
\154	l	*l*	l	*l*	l	l	l	l	↕		⌈	\154	108
\155	m	*m*	m	*m*	m	m	m	m	⇕		⌉	\155	109
\156	n	*n*	n	*n*	n	n	n	n	\		{	\156	110
\157	o	*o*	o	*o*	o	o	o	o	≀		}	\157	111
\160	p	*p*	p	*p*	p	p	p	p	√		√	\160	112
\161	q	*q*	q	*q*	q	q	q	q	∐		√	\161	113
\162	r	*r*	r	*r*	r	r	r	r	∇		√	\162	114
\163	s	*s*	s	*s*	s	s	s	s	∫		√	\163	115
\164	t	*t*	t	*t*	t	t	t	t	⊔		√	\164	116
\165	u	*u*	u	*u*	u	u	u	u	⊓		√	\165	117
\166	v	*v*	v	*v*	v	v	v	v	⊑		⌜	\166	118
\167	w	*w*	w	*w*	w	w	w	w	⊒		‖	\167	119
\170	x	*x*	x	*x*	x	x	x	x	§		↑	\170	120
\171	y	*y*	y	*y*	y	y	y	y	†		↓	\171	121
\172	z	*z*	z	*z*	z	z	z	z	‡		◜	\172	122
\173	–	–	{	ı	–	–	{	–	¶		◝	\173	123
\174	—	—	|	ȷ	—	—	|	—	♣		◟	\174	124
\175	˝	˝	}	℘	˝	˝	}	¨	♢		◞	\175	125

Oct	CMR10	CMTI10	CMTT10	CMMI10	CMU10	CMSS10	CMTEX10	CMFF10	CMSY10	LASY10	CMEX10-Baseline		Oct	Dec
\176	˜	˜	~	$\vec{}$	˜	˜	~	˜	♡			⇑	\176	126
\177, \304	¨	¨	¨	⌢	¨	¨	∫	¨	♠		⇓		\177, \304	127, 196
